# Scanning near-field optical spectroscopy and carrier transport based analysis in mesoscopic regions for two-dimensional semiconductors

**DOI:** 10.1038/s41598-022-13492-8

**Published:** 2022-06-20

**Authors:** Anri Sakurai, Kohei Iwamoto, Yoshihiko Miwa, Hirokazu Hori, Akira Ishikawa, Kazuharu Uchiyama, Kiyoshi Kobayashi, Katsumi Kishino, Masaru Sakai

**Affiliations:** 1 Department of Science and Advanced Materials, University of Yamanashi, 4-3-11 Takeda, Kofu, Yamanashi 400-8511 Japan; 2 Natural Science Laboratory, Toyo University, 5-28-20 Hakusan, Bunkyo-ku, Tokyo 112-8606 Japan; 3 Sophia Nanotechnology Research Center, Sophia University, 7-1 Kioi-cho, Chiyoda-ku, Tokyo 102-8554 Japan

**Keywords:** Characterization and analytical techniques, Imaging techniques, Scanning probe microscopy, Nanophotonics and plasmonics

## Abstract

The measurements of photoexcited transport in mesoscopic regimes reveal the states and properties of mesoscopic systems. In this study, we focused on direct measurements of electromagnetic energy transports in the mesoscopic regions and constructed a scanning tunnelling microscope-assisted multi-probe scanning near-field optical microscope spectroscopy system. After producing an emission energy map through a single-probe measurement, two-probe measurement enables us to observe and analyse carrier transport characteristics. It suggests that exciton generation and transport in the mesoscopic region of semiconductors with quantum structure changes, such as the bias of dopant, affect the excited carrier emission recombination process. The measured probability density of the carrier transported with quantum effects can be used for applications in natural intelligence research by combining it with the analysis using tournament structures. Our developed measurement and analysis methods are expected to clarify the details of carrier's behaviour in the mesoscopic region in various materials and lead to applications for novel optoelectronic devices.

## Introduction

It is complex to handle the theoretical mesoscopic region because of its complicated quantum and classical behaviours. However, this complexity can produce unprecedented functionalities. Therefore, it is essential to develop experimental methods to evaluate the states and properties of mesoscopic systems in detail.

Scanning probe microscopy (SPM) is an instrument used to approach mesoscopic regions. It can change the carrier density in a sample at the nanometer-order using a sharpened probe. It can also measure the density locally.

Scanning near-field optical microscope (SNOM) is an effective SPM used to study the optical excitation and transport of carriers in mesoscopic regions of semiconductors to explain the behaviour of carriers in quantum structures^1–3^. SNOM can set the initial state by carrier generation with local optical excitation (Illumination mode; I-mode) or measure the final state by carrier detection with local density of carrier using a probe by detecting signals related to the sample state such as the spectrum from the local state (Collection mode; C-mode). For example, Kaneda et al. reported the diffusion of carriers generated in InGaN crystals using SNOM^4^. The advantage of exchanging information with the sample using the optical near-field is that one can directly see the transport of the generated carriers inside the sample in a mesoscopic region. It means that, SNOM can measure the change in the density of carriers in the mesoscopic region using light.

Increasing the number of probes enables us to visualise the carrier transport phenomena in the sample. The technique of multiple probe measurements has been evolved in recent years. To the best of our knowledge, several examples of local excitation and local observation by SNOM using two probes have been reported. This includes the measurement of surface plasmon polariton propagation properties^5–9^ and carrier diffusion during local excitation at semiconductor quantum structure^4,10–12^. As stated above, SNOM measurements with multiple probes allow local optical excitation and local detection of carriers simultaneously. It enables the visualisation of the behaviour of moving excited carriers inside the sample and the local density of states through a spectrum. As a probability density, the quantum measurement of carrier transport can be applied to natural intelligence research by combining it with tournament structure analysis^13^.

In this study, we focused on the direct measurement of carriers as electromagnetic energy transport in the mesoscopic region, which is the basis of signal and information transmission. If the excited carriers move only through the sample from the light source to the photodetector, we call this the 'near-field optical transport process'. In this process, the local carrier transport is connected with the electromagnetic wave energy transport inside the semiconductor quantum structure. Therefore, an effort was made to detect the electromagnetic energy transport by observing the excited transport. We constructed a scanning tunnelling microscope (STM) assisted multi-probe (M-probe) SNOM spectroscopy system that enables local excitation injection at arbitrary positions and local emission spectroscopy by scanning the other probe in the region of the excitation point. Consequently, we obtained two-dimensional maps of local energy and density of carrier on the nanometre to micrometre scale.

### M-probe SNOM

**Figure 1:**
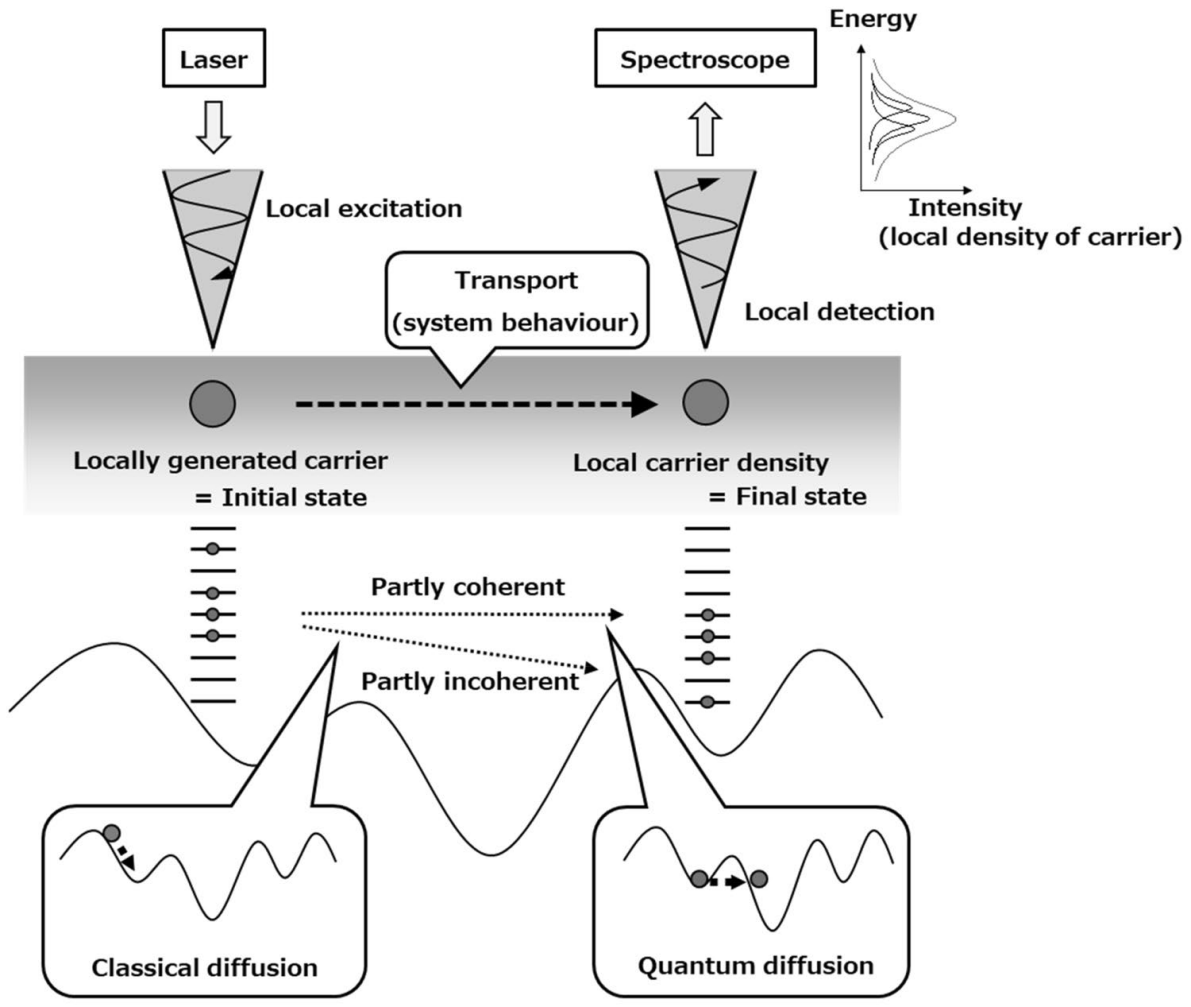
Schematic image of the theory of the two-probe measurement. Excited carriers generated by local excitation at arbitrary points using a sharpened probe diffuse in the sample. The detection of carriers after transportation is performed locally using an observation probe and take each spectrum. In this way, the local carrier density of the carriers diffused in the sample can be directly measured.

Figure 1 shows a schematic image of the two-probe measurement theory. In classical transport, carriers are diffused due to the influence of environmental systems, such as the slope of the material potential. In contrast, in quantum behaviour, we need to consider that carriers carry on a non-clear route of the environmental system, e.g. excitation transfer between resonant levels formed between different quantum dots^14,15^ and excitation transfer through coherent-phonon-assisted under external magnetic fields at semiconductor nanostructures^16,17^. In the mesoscopic system, a composite of classical (i.e. incoherent) and quantum (i.e. coherent), the influence of the environmental system on the carrier and the change in the state of the system by observation should occur simultaneously. However, we cannot directly observe the state of the environmental system. Therefore, this study suggests an approach to predict the influence of the environmental system for carriers using two-probe measurements.

In two-probe measurement, it is possible to determine the initial and final states by each probe. The expected value of excitation, transport, and recombination in the sample is obtained as the emission spectrum using local spectroscopy. The spectrum of observation results can be regarded as being made up of the initial and final states and transport operators. Therefore, in two-probe measurement, the effect of the environmental system (sample) can be obtained by detecting the result of the carrier excited locally through the probe moving to another place in the sample under the influence of the environmental system.

**Figure 2:**
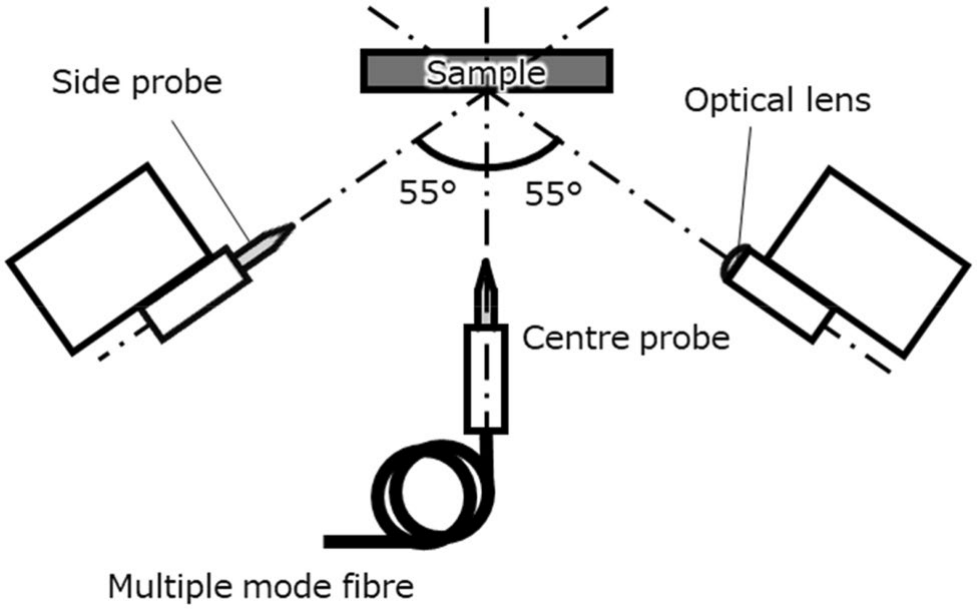
Schematic image of sample and probe units. The centre probe (C-probe) is connected to a multiple-mode optical fibre. Both side attachments (side probe (S-probe) or classical lens) is set at an angle of 55° to the C-probe.

The developed M-probe SNOM is based on STM (UNISOKU USM-1400), and the optical fibre centre probe (C-probe) is placed perpendicular to the sample surface (Fig. 2). The C-probe is connected to a 100 μm core optical fibre inside the M-probe SNOM, enabling direct optical connection outside the chamber. Figure 2 shows that the two side attachment units are placed at angles of 55° to the C-probe. Each side attachment can be selected as either a probe (called the side probe; S-probe) or an optical lens. The system can also be used for measurements at room temperature, 77 K and 4 K.

## Experiment

### Sample and experimental setup

In this paper, we report photoluminescence (PL) spectroscopy of InGaN/GaN multiple quantum wells (MQWs) using the M-probe SNOM. In the InGaN quantum structure, the excited carriers diffuse into the potential energy with fluctuations determined by the composition of indium^18^. However, the details of the diffusion path and process are not fully explored.

**Figure 3:**
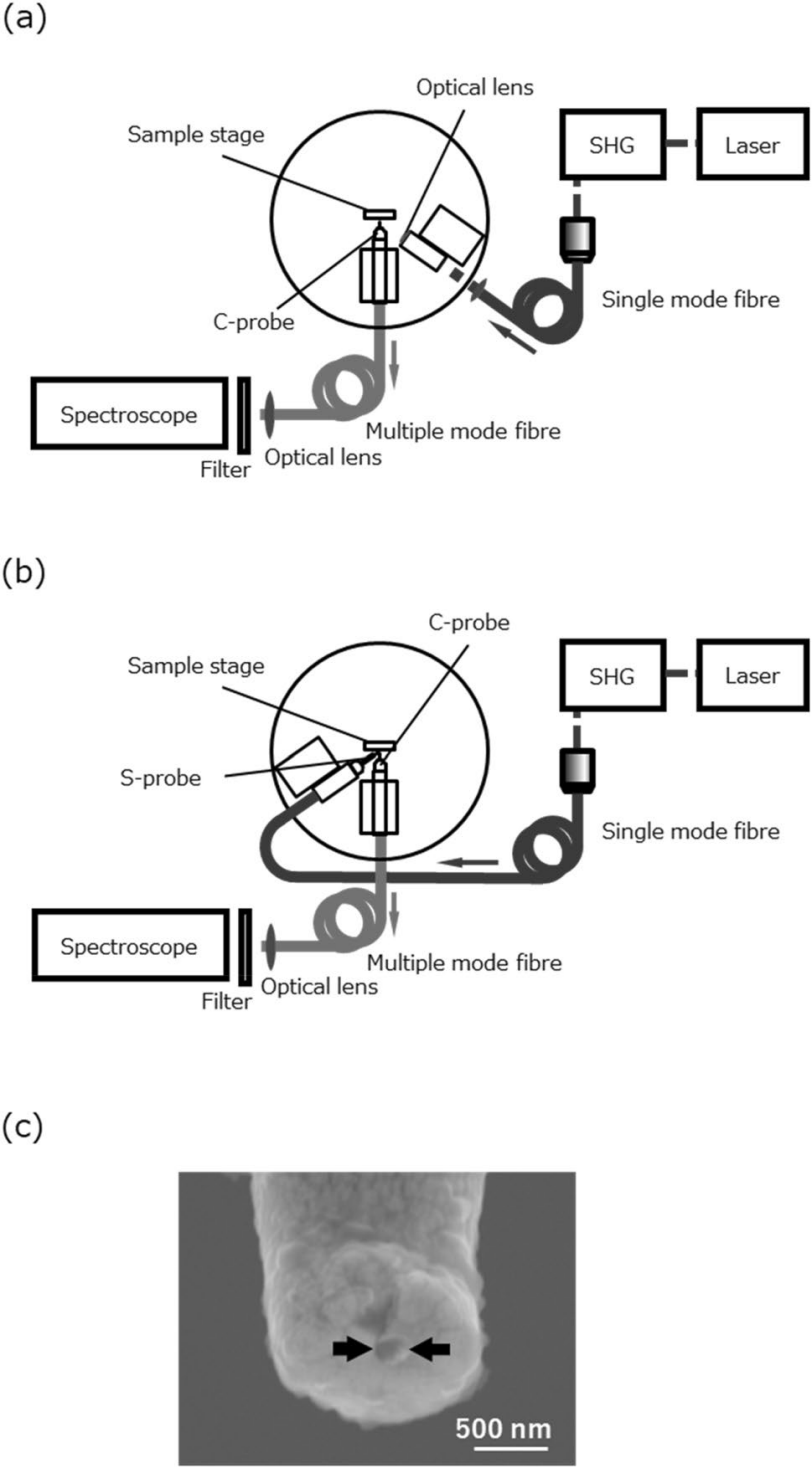
(**a**) Schematic image of the C-mode operation setup. The excitation was conducted with an optical lens on the side attachment. (**b**) Schematic image of the two-probe measurement setup. The excitation was conducted with S-probe. (**c**) A scanning electron microscope image of a typical aperture of C-probe.

Figure 3a,b show schematic of measurement systems. The light source is a frequency-doubled Ti:Sapphire laser (Coherent Mira 900-P; centre wavelength: 800 nm; pulse width: 3 ps; repetition rate: 76 MHz), i.e. a 400-nm pulse laser. The emission from the sample detected by the C-probe is measured spectroscopically using a spectrometer (Princeton Instruments SP-2300i; focal length: 30 cm; grating grooves: 600/mm) with a Si-CCD detector cooled by liquid nitrogen (Roper Scientific Spec-10) through a multimode optical fibre. In the C-mode operation using one probe (Fig. 3a), the sample was excited through a classical optical lens of a diameter of 10 mm and focal length of 15 mm on the side attachment. The emission from the sample was observed locally by the C-probe. On the other hand, an S-probe was used for local excitation instead of the lens on the side attachment in the two-probe measurement (Fig. 3b).

Figure 3c shows a scanning electron microscope image of an aperture-type optical fibre probe tip used as the C-probe. The optical fibre probe was formed pencil shape with a tip angle of about 23° by chemical etching using the meniscus method with hydrofluoric acid^11,18–21^. The probes were coated by Au with a thickness of about 100 nm by sputtering. Consequently, a small aperture was created by pounding the probe apex against a sapphire substrate. In this experiment, we used an aperture-type optical fibre probe with an aperture diameter of about 100 nm for measurement at a spatial resolution of 100 nm.

### Procedure

**Figure 4:**
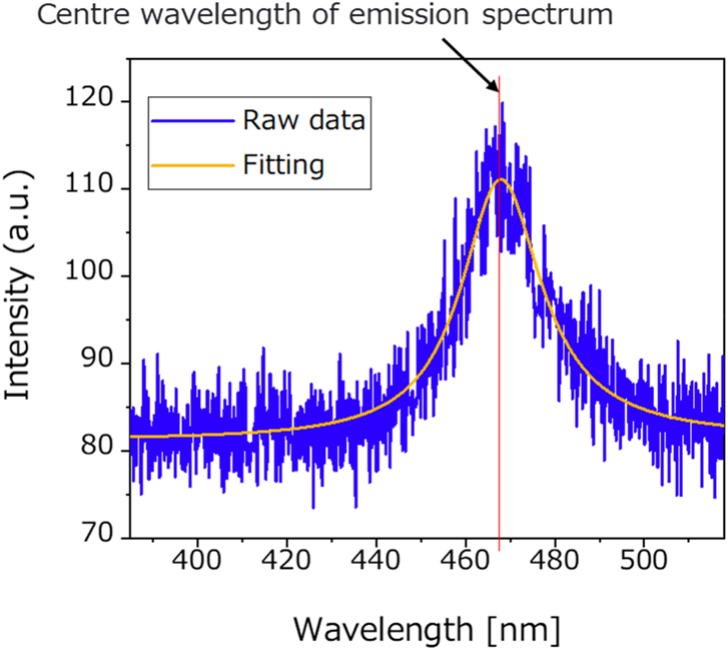
Typical raw spectrum data and fitting curve fitted by Lorentzian.

First, to investigate the optical properties of the sample, local PL spectroscopy was performed by C-mode operation with C-probe at an area of 6 μm × 6 μm at 100 nm intervals, i.e. 3600 points. The spot size of excitation from the far-field is approximately 18 μm in diameter. The centre of the scan area was aligned with the point of greatest excitation intensity (the centre of the spot). This is because the spot size is greater than the scan area and covers all region, the scanning area is completely excited during the C-mode operation. Figure 4 shows a typical local PL spectrum. As shown in Fig. 4, the PL spectrum at each point was fitted by a Lorentzian curve to determine the emission centre wavelength of the emission spectrum. The integral intensity map is created by mapping the integrated intensity of the spectrum over the entire wavelength range of each point. After the C-mode operation, we performed a two-probe measurement. The local PL spectroscopy was conducted at an area of 4.1 μm × 4.1 μm at 100 nm intervals.

**Figure 5:**
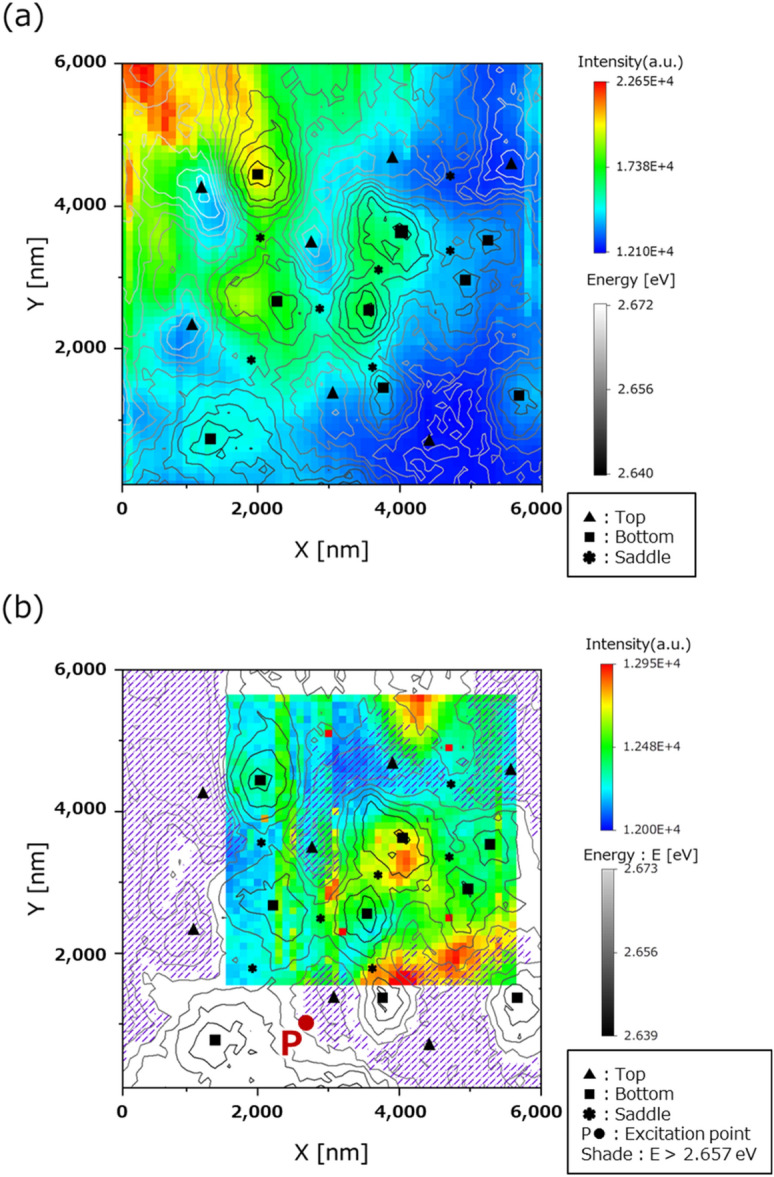
(**a**) Combined image of the energy contour map superimposed on the integral intensity map, which was produced by the C-mode operation result. (**b**) Energy contour map produced by the C-mode operation result superimposed on the integral intensity map of the two-probe measurement. Markers on the map show the characteristic point. (▲: mountain-like potential, ■: valley-like potential, *: saddle-like potential, which is an unstable point). The shaded area has higher energy (> 2.657 eV) than the local excitation point (point P: ●) by S-probe.

An energy contour map was created based on the central energy of the emission at each point obtained by the C-mode operation. Characteristic potential shapes were extracted, (Fig. 5) in grey scale. In Fig. 5, the triangle mark (▲) and the square mark (■) denotes the top and bottom of potential energy, respectively. Additionally, the star mark (*) denotes a saddle point (an unstable point) of the potential energy. Figure 5a is a map that shows a result of the C-mode operation. This map is an energy contour map superimposed on the integrated intensity map. Figure 5b is a map that an energy contour created by the C-mode operation superimposed on the integrated intensity map obtained using the two-probe measurement. The point P(●) marked in Fig. 5b represents the local excitation point using the S-probe. The shaded area in Fig. 5b denotes the area with high potential energy than the local excitation point (point P: ●), 2.657 eV.

### Results and discussion

Carriers excited in semiconductor crystals diffuse, and recombine at each point. The distribution of energy and emission intensity at each point can be determined by examining the spectrum at each point. If this is arranged according to the measurement, the energy and emission intensity distributions throughout the measurement range are plotted. The results obtained in this way are depicted in Fig. 5. As shown in Fig. 5a, the emission intensity in the bottom regions is higher than that in the surrounding hill region. That indicates that carriers generated in the semiconductor quantum well tend to be collected at the bottoms, i.e. the stable points. This tendency is also observed in Fig. 5b. This means that the excited carriers produced at point P went down to the low-energy area as they diffused. Furthermore, in Fig. 5a,b, there is a tendency that in the area of widely spaced (slope is gentle) at the contours are higher intensity. This can be attributed to the increase in time taken by carriers to migrate through the area and the probability of recombination owing to the gradual slope.

Figure 5b shows that in the two-probe measurement, the excited carriers generated at point P seem to diffuse in a way that detours the region of energy higher than point P. It seems that the existence of saddle-shaped potential influences the path selection of the spreading carrier. Therefore, the generated excited carriers are expected to branch at the saddle-shaped potential on the diffusion path and diffuse toward the stable point. This may cause a difference in emission intensity, even at adjacent valley-shaped potentials. This branch will affect the results of C-mode operation and two-probe measurements. The diffusion pathways are considered to be influenced by the energy of the local excitation point and the surrounding energy topography. It is because most carriers diffuse affected by the energy topography. As a result, it assumes that even in the same measurement range, if the local excitation point changes, the spectra obtained at each point after diffusion and the diagrams obtained to change. Therefore, it can consider that although Fig. 5b is the result when the local excitation point is P, the figure also changes when the excitation point varies.

**Figure 6:**
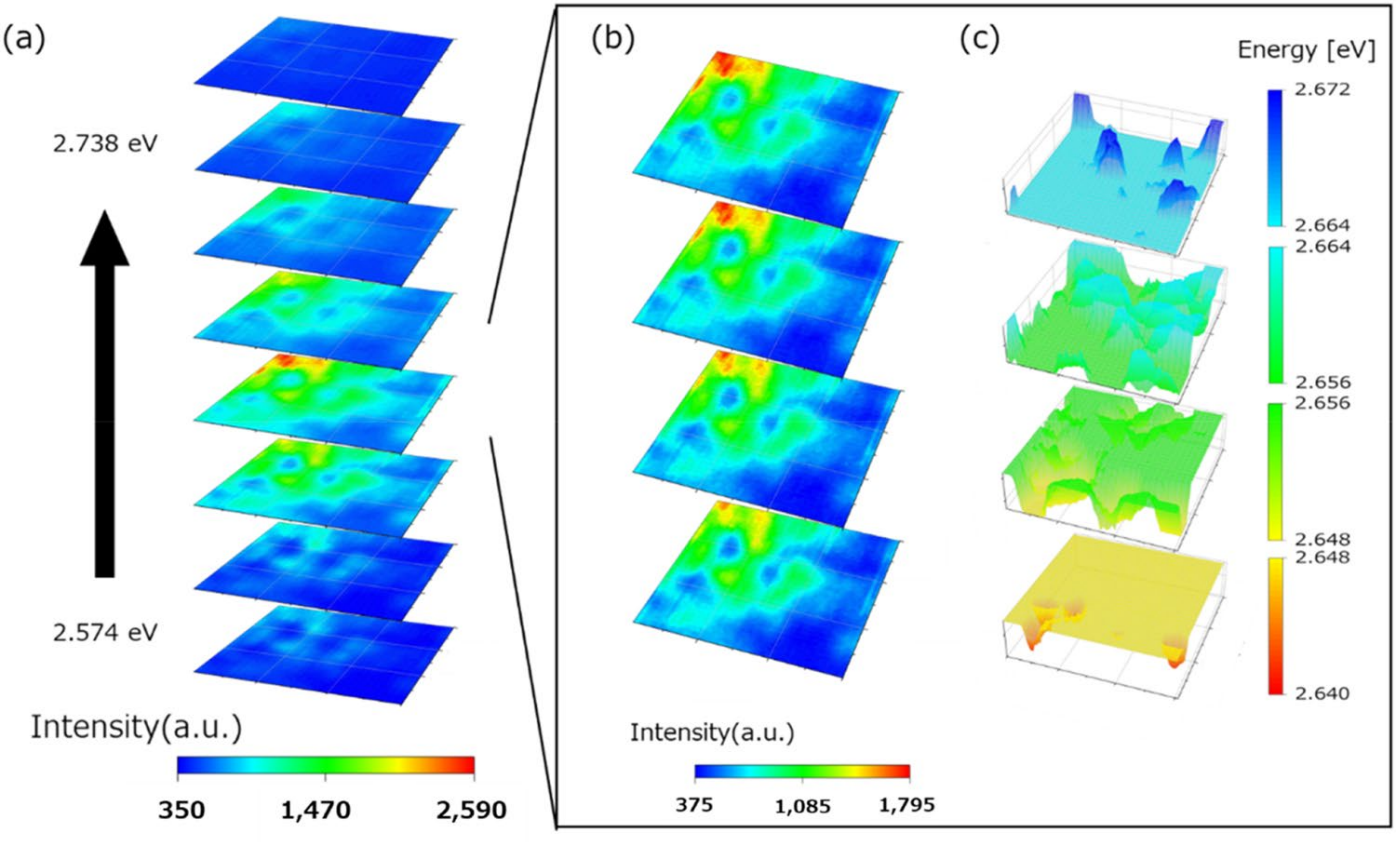
Sliced the integral intensity map obtained from the C-mode operation. (**a**) Sliced every 21 meV map of the main emission energy range. (**b**) More finely (every 8 meV) sliced map within the range of change in the emission centre energy. (**c**) Slices of the three-dimensional emission centre wavelength map. Each slice of (**b**) and (**c**) has the same energy range.

**Figure 7:**
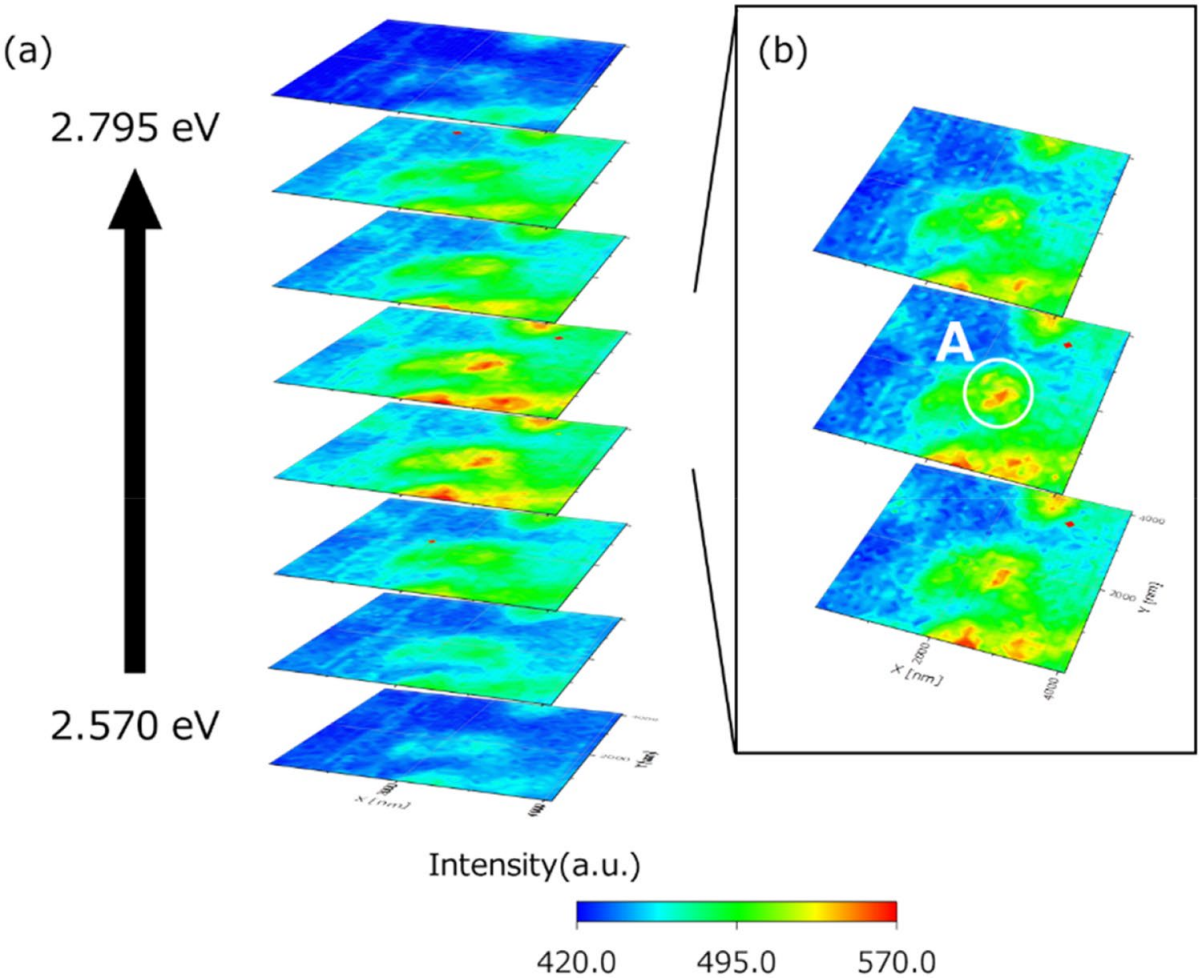
Sliced maps of the integral intensity map, obtained from the two-probe measurement. (**a**) Sliced map of the main emission energy range every 28 meV. (**b**) More finely (every 11.5 meV) sliced map within the range of change in the emission centre energy of (**a**).

The integral intensity maps obtained from the C-mode operation and two-probe measurements were sliced every 21 and 28 meV into a wide range of energies, which were the main emission ranges, as shown in Figs. 6a and 7a, respectively. The emission state of each energy range can be observed when the emission intensity is sliced by energy ranges. In the classical diffusion of excited carriers, they are conceivable to diffuse and emit according to the potential structure of the sample. Therefore, sliced integral emission intensity maps are expected to change continuously in response to the energy change. However, suppose there is a sudden discontinuity in the middle of the energy change, e.g. an island-like emission intensity distribution that is not caused by the potential structure. In that case, it may be due to a phenomenon different from the classical behaviour. These diffusion processes are assumed to have quantum behaviours, such as carrier transport through phonon^22^. The results of the C-mode operation (Fig. 6a) show that the centre of the luminescence is located at the lower energy levels, indicate that the generated excited carriers are relaxed and recombined at the lower energy level. Since slicing over a wide range of energies may overlook changes, we sliced every 8 meV finely over the energy range of the fluctuation of the central wavelength of the emission (Fig. 6b). Figure 6c shows a three-dimensional representation of the energy map at each range corresponding to Fig. 6b. By creating a slice of the three-dimensional potential energy and a slice of the emission intensity, we can intuitively see the contrast between the topography and the emission in each range. The results did not show any clear changes that could be considered as quantum behaviours. A discrepancy with the set analysis conditions (e.g. we need a more finely divided slice) may have prevented the detected transition from being visualised. Further experiments at different conditions are needed to verify the quantum behaviours of the results.

In the two-probe measurements, there is no clear bias towards one side but there is also no simple symmetry. In this analysis, shown in Fig. 7a, the area of higher intensity is larger at the lower image that at the highest image. However, when comparing the second figure from the top and bottom, the upper figure has higher intensity overall. Furthermore, as shown in the region A in Fig. 7b, which is a more detailed (every 11.5 meV) slice of the region of Fig. 7a, where the light emission is strong, it seems that it changes discontinuously from the upper and lower figures. It is considered that these are affected by the ease of formation of energy levels in this sample, excited carriers’ generation conditions, transport pathways, and more. The excited carrier generation and transportation conditions are expected to affect the excited carrier state at each point and be reflected in the emission spectrum obtained by emission recombination. Additionally, as shown in Figs. 5b and 7, there is a region with the same (or higher) energy level as the point P, which is high emission intensity. The carrier transfer to such regions may be due to classical and quantum diffusions. It is considered that the emission intensity distribution changed even when the measurements were made in the same measurement area in Figs. 6 and 7. Therefore, further experiments (e.g. experiments at low temperature) and analyses are required to obtain more information.

## Conclusion

This study reported the direct measurement of electromagnetic energy transport in the mesoscopic region. We suggested the analysis method to observe the mesoscopic scale behaviour of carriers, which is the basis of signal and information transmission. The M-probe SNOM assisted by STM was developed for the first time, and C-mode operation and two-probe measurements were conducted in the same area. Based on the local spectra of these measurements, the centre emission energy and integral emission intensity maps were constructed to discuss the luminescence characteristics of the samples. The two-probe measurement enables us to observe the near-field optical excitation transport. It means that the effect of the environmental system can be obtained by detecting locally the result of the carrier excited by the probe moving to another place in the sample under the influence of the environmental system.

The emission intensity shows a distribution due to the topography of energy potential. Additionally, the carriers excited locally by the probe appear to diffuse, avoiding the energy region higher than the excitation point. The saddle-points in the potential play an essential role in determining the diffusion path. Furthermore, the emission intensity partly depends on the slope of potential energy. This difference indicates that the probability of radiative recombination is higher and lower at the gentle and steep slopes in the potential energy, respectively.

The C-mode operation results show that the emission centre is located at the low energy side by slicing the integral intensity maps into energy ranges. This suggests that the generated excited carriers are relaxed and recombine at lower energy levels. At the two-probe measurement's result, there was no clear bias to one side and no simple symmetry. There were regions that showed a discontinuous change with respect to the energy change. This is because of the ease of formation of energy levels in the sample and the conditions under which the excitation carriers are generated, which determine the state of the excitation carriers at each point. Moreover, these are reflected in the emission spectra obtained by radiative recombination.

Our measurement and analysis methods are expected to clarify the details of the carrier behaviour in the mesoscopic region in various materials. As a probability density, the quantum measurement of carrier transport can be used in natural intelligence research by combining it with the tournament structure analysis. The elucidation of the details of the carrier behaviour in the mesoscopic region can also be applied to novel devices.
